# *trans*-Dibromidobis(triphenyl­phosphine-κ*P*)palladium(II) chloro­form monosolvate

**DOI:** 10.1107/S1600536809027408

**Published:** 2009-07-18

**Authors:** Kong Mun Lo, Seik Weng Ng

**Affiliations:** a Department of Chemistry, University of Malaya, 50603 Kuala Lumpur, Malaysia

## Abstract

The Pd^II^ atom in the title compound, [PdBr_2_{P(C_6_H_5_)_3_}_2_]·CHCl_3_, lies on a twofold rotation axis and is coordinated in a distorted square-planar geometry by two P atoms from two triphenyl­phosphine ligands and by two Br atoms in a *trans* arrangement. The chloro­form solvent mol­ecule is equally disordered about another twofold rotation axis.

## Related literature

For isostructural PdI_2_(PPh_3_)_2_·CHCl_3_, see: Kubota *et al.* (1991 ▶). For the other solvates of PdBr_2_(PPh_3_)_2_, see: Crawforth *et al.* (2005 ▶); Rodríguez *et al.* (2007 ▶); Stark & Whitmire (1997 ▶).
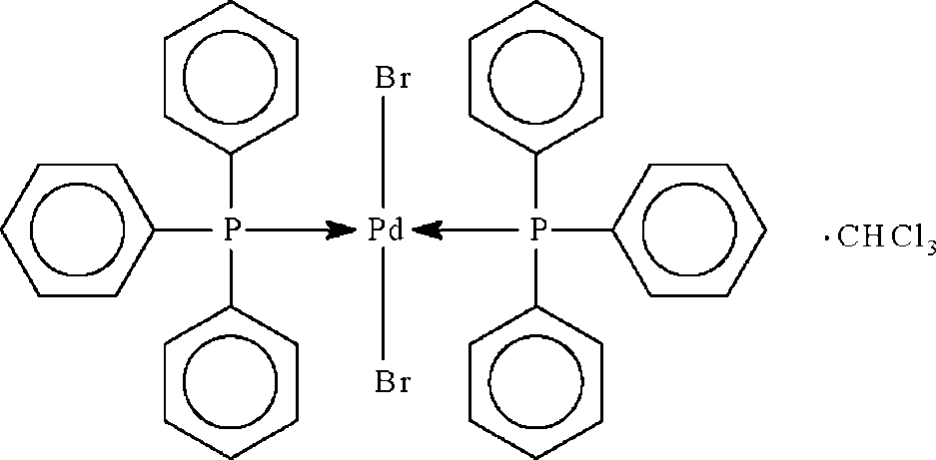


## Experimental

### 

#### Crystal data

[PdBr_2_(C_18_H_15_P)_2_]·CHCl_3_
*M* *_r_* = 910.13
Monoclinic, 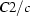

*a* = 12.2314 (2) Å
*b* = 14.4754 (2) Å
*c* = 20.1653 (3) Å
β = 92.477 (1)°
*V* = 3567.02 (9) Å^3^
*Z* = 4
Mo *K*α radiation
μ = 3.10 mm^−1^
*T* = 153 K
0.30 × 0.25 × 0.20 mm

#### Data collection

Bruker SMART APEX CCD diffractometer
Absorption correction: multi-scan (*SADABS*; Sheldrick, 1996 ▶) *T*_min_ = 0.456, *T*_max_ = 0.576 (expected range = 0.426–0.538)
16862 measured reflections
4110 independent reflections
3266 reflections with *I* > 2σ(*I*)
*R*_int_ = 0.018

#### Refinement

*R*[*F* ^2^ > 2σ(*F* ^2^)] = 0.023
*wR*(*F* ^2^) = 0.061
*S* = 1.02
4110 reflections
222 parameters
24 restraints
H-atom parameters constrained
Δρ_max_ = 0.47 e Å^−3^
Δρ_min_ = −0.36 e Å^−3^

### 

Data collection: *APEX2* (Bruker, 2007 ▶); cell refinement: *SAINT* (Bruker, 2007 ▶); data reduction: *SAINT*; program(s) used to solve structure: *SHELXS97* (Sheldrick, 2008 ▶); program(s) used to refine structure: *SHELXL97* (Sheldrick, 2008 ▶); molecular graphics: *X-SEED* (Barbour, 2001 ▶); software used to prepare material for publication: *publCIF* (Westrip, 2009 ▶).

## Supplementary Material

Crystal structure: contains datablocks global, I. DOI: 10.1107/S1600536809027408/hy2209sup1.cif

Structure factors: contains datablocks I. DOI: 10.1107/S1600536809027408/hy2209Isup2.hkl

Additional supplementary materials:  crystallographic information; 3D view; checkCIF report

## Figures and Tables

**Table 1:** Selected bond lengths (Å)

Pd1—P1	2.3360 (5)
Pd1—Br1	2.4277 (2)

**Fig. 1.**
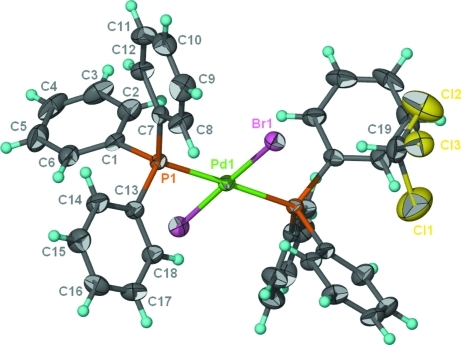
Molecular structure of the title compound. Displacement ellipsoids are drawn at the 70% probability level.

## supplementary crystallographic information

### Experimental

Commercially available dark-brown bis(triphenylphosphine)palladium dichloride (0.70 g, 1 mmol) and 4-dimethylaminopyridinium hydrobromide perbromide (0.36 g, 1 mmol) were heated in an ethanol/chloroform mixture (1:1 v/v, 100 ml) for an hour. The solution was filtered and a small amount of deep yellow crystals were isolated along with some dark brown material.

### Refinement

H atoms were placed at calculated positions (C–H = 0.95 and 1.00 Å) and treated as riding on their parent atoms, with *U*_iso_(H) = 1.2*U*_eq_(C). The chloroform molecule is disordered about a twofold rotation axis, and was allowed to refine off the symmetry element as a whole molecule of 0.5 site occupancy. The three C—Cl distances were restrained to within 0.01 Å of each other, as were the Cl···Cl distances. The anisotropic displacements of the Cl atoms were restrained to be nearly isotropic.

### Crystal data

**Table:**

[PdBr_2_(C_18_H_15_P)_2_]·CHCl_3_	*F*(000) = 1800
*M**_r_* = 910.13	*D*_x_ = 1.695 Mg m^−^^3^
Monoclinic, *C*2/*c*	Mo *K*α radiation, λ = 0.71073 Å
Hall symbol: -C 2yc	Cell parameters from 6987 reflections
*a* = 12.2314 (2) Å	θ = 2.4–28.3°
*b* = 14.4754 (2) Å	µ = 3.10 mm^−^^1^
*c* = 20.1653 (3) Å	*T* = 153 K
β = 92.477 (1)°	Prism, brown
*V* = 3567.02 (9) Å^3^	0.30 × 0.25 × 0.20 mm
*Z* = 4	

### Data collection

**Table:**

Bruker SMART APEX CCD diffractometer	4110 independent reflections
Radiation source: fine-focus sealed tube	3266 reflections with *I* > 2σ(*I*)
graphite	*R*_int_ = 0.018
φ and ω scans	θ_max_ = 27.5°, θ_min_ = 2.0°
Absorption correction: multi-scan (*SADABS*; Sheldrick, 1996)	*h* = −15→15
*T*_min_ = 0.456, *T*_max_ = 0.576	*k* = −18→18
16862 measured reflections	*l* = −26→26

### Refinement

**Table:**

Refinement on *F*^2^	Primary atom site location: structure-invariant direct methods
Least-squares matrix: full	Secondary atom site location: difference Fourier map
*R*[*F*^2^ > 2σ(*F*^2^)] = 0.023	Hydrogen site location: inferred from neighbouring sites
*wR*(*F*^2^) = 0.061	H-atom parameters constrained
*S* = 1.02	*w* = 1/[σ^2^(*F*_o_^2^) + (0.0263*P*)^2^ + 6.7144*P*] where *P* = (*F*_o_^2^ + 2*F*_c_^2^)/3
4110 reflections	(Δ/σ)_max_ = 0.001
222 parameters	Δρ_max_ = 0.47 e Å^−^^3^
24 restraints	Δρ_min_ = −0.36 e Å^−^^3^

### Fractional atomic coordinates and isotropic or equivalent isotropic displacement parameters (Å^2^)

**Table:**

	*x*	*y*	*z*	*U*_iso_*/*U*_eq_	Occ. (<1)
Pd1	0.5000	0.250839 (14)	0.2500	0.01811 (6)	
Br1	0.322982 (17)	0.253392 (15)	0.299982 (11)	0.02692 (7)	
P1	0.59326 (4)	0.25342 (3)	0.35355 (3)	0.01868 (11)	
C1	0.70341 (17)	0.16892 (14)	0.35728 (10)	0.0226 (4)	
C2	0.6778 (2)	0.08037 (16)	0.33385 (12)	0.0324 (5)	
H2	0.6064	0.0675	0.3159	0.039*	
C3	0.7560 (2)	0.01137 (17)	0.33665 (13)	0.0418 (6)	
H3	0.7378	−0.0492	0.3217	0.050*	
C4	0.8602 (2)	0.0307 (2)	0.36119 (13)	0.0465 (7)	
H4	0.9142	−0.0165	0.3624	0.056*	
C5	0.8869 (2)	0.1177 (2)	0.38400 (14)	0.0443 (7)	
H5	0.9591	0.1303	0.4008	0.053*	
C6	0.80823 (19)	0.18742 (17)	0.38251 (11)	0.0306 (5)	
H6	0.8264	0.2473	0.3987	0.037*	
C7	0.51852 (17)	0.22807 (14)	0.42793 (10)	0.0218 (4)	
C8	0.4458 (2)	0.29338 (17)	0.45150 (12)	0.0339 (5)	
H8	0.4357	0.3504	0.4287	0.041*	
C9	0.3886 (2)	0.2759 (2)	0.50751 (13)	0.0405 (6)	
H9	0.3397	0.3212	0.5231	0.049*	
C10	0.4014 (2)	0.19368 (19)	0.54117 (12)	0.0369 (6)	
H10	0.3613	0.1820	0.5796	0.044*	
C11	0.4724 (2)	0.12875 (17)	0.51881 (12)	0.0356 (6)	
H11	0.4821	0.0721	0.5421	0.043*	
C12	0.53046 (19)	0.14535 (15)	0.46205 (11)	0.0292 (5)	
H12	0.5787	0.0995	0.4466	0.035*	
C13	0.65111 (17)	0.36690 (14)	0.37174 (10)	0.0219 (4)	
C14	0.6954 (2)	0.38791 (16)	0.43498 (11)	0.0314 (5)	
H14	0.6938	0.3432	0.4694	0.038*	
C15	0.7417 (2)	0.47408 (16)	0.44744 (12)	0.0352 (6)	
H15	0.7720	0.4881	0.4904	0.042*	
C16	0.7439 (2)	0.53955 (15)	0.39760 (12)	0.0316 (5)	
H16	0.7776	0.5978	0.4060	0.038*	
C17	0.6973 (2)	0.52028 (15)	0.33583 (12)	0.0315 (5)	
H17	0.6968	0.5660	0.3020	0.038*	
C18	0.65112 (18)	0.43408 (14)	0.32274 (11)	0.0258 (5)	
H18	0.6193	0.4211	0.2799	0.031*	
Cl1	0.0544 (6)	0.4068 (4)	0.1875 (3)	0.083 (2)	0.50
Cl2	−0.0249 (2)	0.23500 (12)	0.23506 (13)	0.0769 (8)	0.50
Cl3	−0.0654 (5)	0.4028 (2)	0.3067 (2)	0.0454 (8)	0.50
C19	0.0330 (4)	0.3413 (3)	0.2596 (2)	0.0423 (13)	0.50
H19	0.1030	0.3321	0.2862	0.051*	0.50

### Atomic displacement parameters (Å^2^)

**Table:**

	*U*^11^	*U*^22^	*U*^33^	*U*^12^	*U*^13^	*U*^23^
Pd1	0.02113 (11)	0.01538 (10)	0.01773 (11)	0.000	−0.00004 (8)	0.000
Br1	0.02633 (11)	0.02758 (12)	0.02707 (12)	−0.00200 (9)	0.00362 (8)	0.00051 (9)
P1	0.0216 (2)	0.0161 (2)	0.0182 (2)	0.0013 (2)	−0.00033 (19)	0.00040 (19)
C1	0.0282 (11)	0.0208 (10)	0.0190 (10)	0.0061 (8)	0.0032 (8)	0.0033 (8)
C2	0.0430 (14)	0.0238 (11)	0.0302 (12)	0.0056 (10)	0.0000 (10)	−0.0027 (9)
C3	0.0663 (19)	0.0268 (12)	0.0327 (13)	0.0174 (12)	0.0091 (13)	0.0022 (10)
C4	0.0561 (18)	0.0461 (16)	0.0382 (15)	0.0334 (14)	0.0129 (13)	0.0146 (12)
C5	0.0313 (13)	0.0581 (18)	0.0434 (15)	0.0148 (12)	0.0005 (11)	0.0121 (13)
C6	0.0291 (12)	0.0335 (12)	0.0292 (12)	0.0037 (9)	0.0019 (10)	0.0050 (9)
C7	0.0223 (10)	0.0233 (10)	0.0194 (10)	−0.0015 (8)	−0.0020 (8)	0.0006 (8)
C8	0.0394 (13)	0.0319 (13)	0.0311 (13)	0.0094 (11)	0.0082 (11)	0.0063 (10)
C9	0.0385 (14)	0.0485 (15)	0.0353 (14)	0.0093 (12)	0.0113 (11)	−0.0042 (12)
C10	0.0363 (13)	0.0515 (16)	0.0234 (12)	−0.0087 (12)	0.0078 (10)	0.0012 (11)
C11	0.0462 (15)	0.0323 (13)	0.0286 (12)	−0.0070 (11)	0.0047 (11)	0.0071 (10)
C12	0.0361 (13)	0.0232 (11)	0.0284 (12)	0.0003 (9)	0.0031 (10)	0.0021 (9)
C13	0.0247 (10)	0.0178 (9)	0.0232 (10)	0.0005 (8)	0.0011 (8)	−0.0023 (8)
C14	0.0429 (14)	0.0257 (11)	0.0253 (11)	−0.0031 (10)	−0.0036 (10)	0.0004 (9)
C15	0.0459 (14)	0.0312 (12)	0.0279 (12)	−0.0034 (11)	−0.0060 (11)	−0.0074 (10)
C16	0.0375 (13)	0.0212 (11)	0.0366 (13)	−0.0056 (10)	0.0062 (10)	−0.0084 (9)
C17	0.0429 (14)	0.0206 (10)	0.0315 (12)	−0.0019 (10)	0.0077 (11)	0.0018 (9)
C18	0.0329 (12)	0.0210 (10)	0.0233 (11)	0.0002 (9)	0.0012 (9)	−0.0008 (8)
Cl1	0.087 (3)	0.103 (3)	0.059 (2)	0.013 (2)	0.0104 (18)	0.0265 (19)
Cl2	0.078 (2)	0.0667 (10)	0.089 (2)	−0.0211 (10)	0.0363 (14)	−0.0134 (10)
Cl3	0.0476 (13)	0.0525 (16)	0.0375 (14)	0.0017 (12)	0.0166 (12)	−0.0003 (12)
C19	0.030 (3)	0.056 (3)	0.041 (3)	0.004 (2)	−0.003 (2)	0.007 (3)

### Geometric parameters (Å, °)

**Table:**

Pd1—P1	2.3360 (5)	C9—C10	1.376 (4)
Pd1—P1^i^	2.3360 (5)	C9—H9	0.9500
Pd1—Br1	2.4277 (2)	C10—C11	1.369 (4)
Pd1—Br1^i^	2.4277 (2)	C10—H10	0.9500
P1—C1	1.819 (2)	C11—C12	1.394 (3)
P1—C13	1.820 (2)	C11—H11	0.9500
P1—C7	1.827 (2)	C12—H12	0.9500
C1—C6	1.385 (3)	C13—C18	1.386 (3)
C1—C2	1.397 (3)	C13—C14	1.397 (3)
C2—C3	1.382 (3)	C14—C15	1.388 (3)
C2—H2	0.9500	C14—H14	0.9500
C3—C4	1.377 (4)	C15—C16	1.383 (3)
C3—H3	0.9500	C15—H15	0.9500
C4—C5	1.375 (4)	C16—C17	1.376 (3)
C4—H4	0.9500	C16—H16	0.9500
C5—C6	1.394 (3)	C17—C18	1.390 (3)
C5—H5	0.9500	C17—H17	0.9500
C6—H6	0.9500	C18—H18	0.9500
C7—C12	1.386 (3)	Cl1—C19	1.764 (5)
C7—C8	1.395 (3)	Cl2—C19	1.756 (4)
C8—C9	1.377 (3)	Cl3—C19	1.800 (5)
C8—H8	0.9500	C19—H19	1.0000
			
P1—Pd1—P1^i^	178.16 (3)	C10—C9—C8	120.8 (2)
P1—Pd1—Br1	92.204 (14)	C10—C9—H9	119.6
P1^i^—Pd1—Br1	87.768 (14)	C8—C9—H9	119.6
P1—Pd1—Br1^i^	87.768 (14)	C11—C10—C9	119.5 (2)
P1^i^—Pd1—Br1^i^	92.204 (14)	C11—C10—H10	120.3
Br1—Pd1—Br1^i^	178.256 (14)	C9—C10—H10	120.3
C1—P1—C13	108.54 (10)	C10—C11—C12	120.3 (2)
C1—P1—C7	103.12 (9)	C10—C11—H11	119.8
C13—P1—C7	102.70 (9)	C12—C11—H11	119.8
C1—P1—Pd1	111.00 (7)	C7—C12—C11	120.7 (2)
C13—P1—Pd1	111.42 (7)	C7—C12—H12	119.7
C7—P1—Pd1	119.22 (7)	C11—C12—H12	119.7
C6—C1—C2	119.5 (2)	C18—C13—C14	119.07 (19)
C6—C1—P1	123.88 (17)	C18—C13—P1	120.10 (16)
C2—C1—P1	116.65 (17)	C14—C13—P1	120.83 (16)
C3—C2—C1	120.4 (2)	C15—C14—C13	120.0 (2)
C3—C2—H2	119.8	C15—C14—H14	120.0
C1—C2—H2	119.8	C13—C14—H14	120.0
C4—C3—C2	119.7 (2)	C16—C15—C14	120.3 (2)
C4—C3—H3	120.1	C16—C15—H15	119.8
C2—C3—H3	120.1	C14—C15—H15	119.8
C5—C4—C3	120.6 (2)	C17—C16—C15	119.9 (2)
C5—C4—H4	119.7	C17—C16—H16	120.0
C3—C4—H4	119.7	C15—C16—H16	120.0
C4—C5—C6	120.2 (3)	C16—C17—C18	120.2 (2)
C4—C5—H5	119.9	C16—C17—H17	119.9
C6—C5—H5	119.9	C18—C17—H17	119.9
C1—C6—C5	119.7 (2)	C13—C18—C17	120.4 (2)
C1—C6—H6	120.2	C13—C18—H18	119.8
C5—C6—H6	120.2	C17—C18—H18	119.8
C12—C7—C8	118.1 (2)	Cl2—C19—Cl1	108.1 (3)
C12—C7—P1	122.38 (17)	Cl2—C19—Cl3	108.1 (3)
C8—C7—P1	119.50 (17)	Cl1—C19—Cl3	107.2 (3)
C9—C8—C7	120.6 (2)	Cl2—C19—H19	111.1
C9—C8—H8	119.7	Cl1—C19—H19	111.1
C7—C8—H8	119.7	Cl3—C19—H19	111.1
			
Br1—Pd1—P1—C1	134.30 (8)	C13—P1—C7—C8	50.5 (2)
Br1^i^—Pd1—P1—C1	−47.44 (8)	Pd1—P1—C7—C8	−73.26 (19)
Br1—Pd1—P1—C13	−104.60 (7)	C12—C7—C8—C9	0.6 (4)
Br1^i^—Pd1—P1—C13	73.65 (7)	P1—C7—C8—C9	−179.9 (2)
Br1—Pd1—P1—C7	14.74 (8)	C7—C8—C9—C10	−0.4 (4)
Br1^i^—Pd1—P1—C7	−167.00 (8)	C8—C9—C10—C11	0.4 (4)
C13—P1—C1—C6	11.4 (2)	C9—C10—C11—C12	−0.6 (4)
C7—P1—C1—C6	−97.0 (2)	C8—C7—C12—C11	−0.9 (3)
Pd1—P1—C1—C6	134.19 (17)	P1—C7—C12—C11	179.67 (18)
C13—P1—C1—C2	−169.57 (17)	C10—C11—C12—C7	0.9 (4)
C7—P1—C1—C2	82.00 (18)	C1—P1—C13—C18	112.87 (18)
Pd1—P1—C1—C2	−46.79 (18)	C7—P1—C13—C18	−138.41 (18)
C6—C1—C2—C3	0.9 (3)	Pd1—P1—C13—C18	−9.6 (2)
P1—C1—C2—C3	−178.16 (19)	C1—P1—C13—C14	−67.5 (2)
C1—C2—C3—C4	−1.6 (4)	C7—P1—C13—C14	41.2 (2)
C2—C3—C4—C5	1.1 (4)	Pd1—P1—C13—C14	169.98 (16)
C3—C4—C5—C6	0.0 (4)	C18—C13—C14—C15	−2.0 (3)
C2—C1—C6—C5	0.2 (3)	P1—C13—C14—C15	178.35 (19)
P1—C1—C6—C5	179.22 (18)	C13—C14—C15—C16	0.2 (4)
C4—C5—C6—C1	−0.7 (4)	C14—C15—C16—C17	1.8 (4)
C1—P1—C7—C12	−17.3 (2)	C15—C16—C17—C18	−2.1 (4)
C13—P1—C7—C12	−130.09 (19)	C14—C13—C18—C17	1.8 (3)
Pd1—P1—C7—C12	106.19 (18)	P1—C13—C18—C17	−178.57 (17)
C1—P1—C7—C8	163.24 (19)	C16—C17—C18—C13	0.2 (4)

Symmetry codes: (i) −*x*+1, *y*, −*z*+1/2.
